# One century later: dissecting genetic effects for looking over old paradigms

**DOI:** 10.3389/fgene.2014.00396

**Published:** 2014-11-12

**Authors:** José M. Álvarez-Castro, Rong-Cai Yang

**Affiliations:** ^1^Department of Genetics, Universidade de Santiago de CompostelaLugo, Spain; ^2^Department of Agricultural, Food and Nutritional Science, University of AlbertaEdmonton, AB, Canada

**Keywords:** genetic effects, mathematical modeling, statistical estimation, genetic architecture, environmental effects

The foundation of genetics as a scientific field at the beginning of the twentieth century was not free from controversy. It meant no resolution that the advocates of the Biometric and the Mendelian schools agreed in one thing: the inheritance laws Mendel inferred by studying meristic (discrete) traits did not seem to be compatible with the findings the biometricians had been reporting for continuous (quantitative) variation since the nineteenth century (see Provine, 1971). For providing conclusive evidence against that paradigm, Fisher (1918) developed the foundations of the mathematical models of genetic effects that remain pertinent today, an endeavor in which he developed statistical tools that soon became broadly used beyond genetics.

The genetic effects comprised the core of that theory, but they were initially implemented in those expressions as parameters neither to be estimated nor to actually take any defined numerical values. The most parsimonious hypothesis about genetic effects at that time proposed that the genetic basis of quantitative traits is dominated by the effects of large numbers of genes at which allele substitutions have very small (infinitesimal) and independent (additive) effects on phenotype. This was eventually called the infinitesimal model (see e.g., Bulmer, 1980). Despite the accumulation of evidences suggesting more complex genetic architectures (e.g., Dobzhansky, 1970), the infinitesimal model proved to be a useful paradigm to guide investigation of practical quantitative genetics.

At the time when mapping genetic architectures has moved out the domains of pure fiction (see e.g., Rifkin, 2012), new possibilities for reassessing the adequacy of the infinitesimal model not only reawaken our thirst of knowledge but shall also enable a leap in applicability. It is thus not surprising to witness an increased research effort in updating mathematical and statistical tools for analysing genetic effects, aiming to typify all possible kinds of genetic architectures and their evolutionary implications. We feel grateful for having been able to gather a stimulating account of that update within the current Frontiers Research Topic Issue on Models and Estimation of Genetic Effects.

In the first work in this volume, Gjuvsland et al. (2013) analyse epistasis in genetic networks by focusing on monotonicity as a (correlated) alternative to additivity. Their approach further illustrates that population-referenced (statistical) and non-population-referenced (physiological, functional) genetic parameters are complementary tools in quantitative genetics analyses. The next work, by Le Rouzic (2014), stresses that the evolutionary implications of epistasis are conditioned on whether the interactions follow patterns. He uses the multilinear model to provide practical tools for the detection of such patterns (particularly, directionality) in real data, as well as conceptual keys for aiding the interpretation of the results.

We then move to imprinting, through a work by Álvarez-Castro (2014), who extends the NOIA model to account for that phenomenon and discusses the mathematical properties of the resulting theory in comparison with previous models of imprinting. Further, general procedures for advanced implementation of models of genetic effects are presented in that work. NOIA is also used by Álvarez-Castro and Yang (2012) in the next communication for clarifying the interpretation of the genetic effects defined as average excesses by Ronald Fisher. The interest raised by the publication of that work in Frontiers in Genetics actually triggered the current Research Topic Issue.

A group of papers follows that explicitly account for the environment. Yang (2014) analyses experimental datasets with non-linear functions and addresses some common constraints of the use of linear models to gene by environment interactions. He shows that even under largely linear genotypic responses, strong gene by environment interactions occur because of differences in positions and effects of quantitative trait loci (QTL) between poor and good environments. Marigorta and Gibson (2014) perform simulation studies to tackle the particularities of genome wide association (GWA) human studies. They show that for a wide range of scenarios, cumulative risk of alleles is highly significant despite the lack of evidence for gene by environment interactions, and that increased phenotypic variance after environmental perturbation lowers the statistical power to detect risk alleles in mixed cohorts. The environment of one species may be conditioned by the genome of another, like in the following study by Kodaman et al. (2014) on host-pathogen interactions. They illustrate how pathogens and their human hosts have interacted and coevolved to reduce antagonism and they endorse such information to be incorporated into genetic models to account for the heterogeneity of disease pathology and to avoid dubious conclusions about disease etiology.

The last two communications offer new insights into statistical issues commonly encountered in QTL mapping and GWA studies. Loredo-Osti (2014) provides a bootstrapping procedure to estimate the *p*-values under the mixed-model framework that is applied to QTL mapping when the mapping population consists of recombinant congenic strains, which overcomes a problem concerning the Type I error that had been pointed out in previous approaches. To conclude our compilation, Dai et al. (2014) address the classic issue of multiple hypothesis tests in the current era of high throughput genomics. They advocate a new (modified Lancaster) procedure that improves the control of the Type I error as compared to the Fisher's combination test as well as to the original Lancaster procedure, whilst maintaining statistical power to detect signals related to biomarkers in pathways.

We also find it worth noting that a couple of interesting works addressing genetic effects have been released during the preparation of this editorial. Wang (2014) provides new developments leading to the same genetic variance decomposition of multiallelic loci under departures from the Hardy-Weinberg proportions that we obtained using NOIA (Álvarez-Castro and Yang, 2011; incidentally, we hereby thank Dr. Wang for pointing out a misprint in one of the values of the applied case we provided in our paper). Varona et al. (2014) also use NOIA for dissecting genetic covariances between individuals in the context of genomic selection. Although this kind of analysis was originally developed under the paradigm of the infinitesimal model, and was specifically designed for accounting for any putative infinitesimal additive genetic signal, it is encouraging that it effectively utilizes innovative models of genetic effects. Finally, we commend the coming publication of a volume devoted to a specific (and important) instance of genetic effects, “Epistasis. Methods and Protocols” Edited by Jason H Moore and Scott M Williams, which can be viewed as a new instalment of the already classical “Epistasis and the Evolutionary Process” (Wolf et al., 2000) and whose author list overlaps with that of this Frontiers Research Topic Issue on Models of Genetic Effects.

We hope the papers in this volume provide a useful compendium of theoretical and statistical developments, data analyses, simulation studies, conceptual contributions and discussion that collectively advance knowledge of genetic architectures and environmental interactions, and their broad implications in evolutionary and population genetics. To better contextualize the consequence of this volume, we recall that the recent Frontiers Specialty Grand Challenge Article of Evolutionary and Population Genetics identifies the integration of genomics, modeling and experimentation as both the most critical challenge and exciting opportunity in advancing our field (Cushman, 2014). We feel that the papers presented in this volume, by showing strong linkages and synergies among modeling, experimentation, genomics and bioinformatics, demonstrate the importance of this kind of integrative research. Updating models of genetic effects is critical to take advantage of the stunning burst of molecular techniques and computing capabilities we are witnessing. Obtaining more general formulations of those models shall enable us to more efficiently characterize genetic architectures and to formulate hypothesis that could better guide experimental and simulation studies. Ultimately, evolutionary and population genetics benefits from the integration of different perspectives, methodologies and scopes of research within it, which in its turn accelerates its integration into a fully-fledged science of evolutionary quantitative genetics.

## Conflict of interest statement

The authors declare that the research was conducted in the absence of any commercial or financial relationships that could be construed as a potential conflict of interest.
